# Impact of Positive-Margin Resection of External Auditory Canal Squamous Cell Carcinoma

**DOI:** 10.3390/cancers15174289

**Published:** 2023-08-27

**Authors:** Noritaka Komune, Ryosuke Kuga, Takahiro Hongo, Daisuke Kuga, Kuniaki Sato, Takashi Nakagawa

**Affiliations:** 1 Department of Otorhinolaryngology, Graduate School of Medical Sciences, Kyushu University, Fukuoka 812-8582, Japan; kuga.ryosuke.310@m.kyushu-u.ac.jp (R.K.); hongo.takahiro.604@m.kyushu-u.ac.jp (T.H.); kusato@ucsd.edu (K.S.); nakagawa.takashi.284@m.kyushu-u.ac.jp (T.N.); 2 Department of Anatomic Pathology, Pathological, Sciences, Graduate School of Medical Sciences, Kyushu University, Fukuoka 812-8582, Japan; 3 Department of Neurosurgery, Graduate School of Medical Sciences, Kyushu University, Fukuoka 812-8582, Japan; kuga.daisuke.169@m.kyushu-u.ac.jp

**Keywords:** external auditory canal, squamous cell carcinoma, temporal bone, surgical margin, skull base surgery

## Abstract

**Simple Summary:**

Squamous cell carcinoma of the external auditory canal (EAC-SCC) is sporadic. Its rarity has impeded the collection of evidence from both clinical and basic research, which has delayed the establishment of a standard treatment guideline. It has been considered that negative-margin resection offers the best prognosis. On the other hand, positive-margin resection is still a major cause of recurrence. However, to date, no research has focused on cases with positive-margin resection or specifically assessed the clinical impact of positive-margin resection of EAC-SCC. Our study emphasizes the importance of achieving a negative-margin resection, and we would provide impetus for elucidating the mechanisms of treatment resistance in cases of positive-margin resection.

**Abstract:**

Background: Positive-margin resection of external auditory canal squamous cell carcinoma (EAC-SCC) is still a major cause of recurrence. The aim of this study is to examine the clinical impact of positive-margin resection of EAC-SCCs. Methods: We retrospectively reviewed 40 surgical cases with en bloc temporal bone resection of EAC-SCC at a tertiary referral center from October 2016 to March 2022. Results: Two-year disease-specific, overall, and disease-free survival rates for all 40 cases reviewed were 85.2%, 88.85%, and 76.96%, respectively. En bloc resection with a negative margin significantly improved patient prognosis (*p* < 0.001). Positive-margin resection was observed in 9/40 cases (22.5%). Insufficient assessment of preoperative images was the cause in two of these cases. Postoperative lymph node metastasis and distant metastasis were observed in cases in which vascular, lymphatic duct or perineural invasion was found on postoperative pathological examination. In addition, three cases in which no vascular, lymphatic duct, or perineural invasion was found exhibited local recurrence during the follow-up period. Of the nine positive-margin resection cases, only two showed no postoperative recurrence. Conclusions: Once positive-margin resections are confirmed, cases might have a high risk of tumor recurrence, even with the addition of postoperative adjuvant chemoradiotherapy.

## 1. Introduction

Squamous cell carcinoma (SCC) of the external auditory canal (EAC) is sporadic [1]. Its rarity has impeded the collection of evidence from both clinical and basic research, which has delayed the establishment of a standard treatment guideline. The widely used modified Pittsburgh staging system appears to be suitable for EAC-SCC, although some authors have proposed a new classification system, which could be further improved [2,3,4]. Despite those, treatment outcomes have improved over time because of the development of a key drug (cisplatin), advances in adjuvant therapy, improvements in team medicine, and enhanced preoperative imaging [5,6,7].

Studies have reported that a negative-margin resection offers the best prognosis, and this finding is globally accepted. Postoperative radiotherapy (RT) is reportedly effective for controlling residual lesions if a positive-margin resection is confirmed [8,9]. However, several groups have reported that a positive-margin resection is a major cause of recurrence and criticize postoperative RT as ineffective [1,10,11,12]. Thus, the clinical impact of a positive-margin resection remains controversial. To date, no research has focused on cases with a positive-margin resection or specifically assessed the clinical impact of a positive-margin resection of EAC-SCC. Our objective here was thus to retrospectively review 40 surgical cases and explore the clinical impact of positive-margin resections of EAC-SCC.

## 2. Materials and Methods

### 2.1. Case Profiles (Table 1)

We retrospectively reviewed 40 surgical cases of en bloc temporal bone resection (TBR) for EAC-SCC (T1: 1 case; T2: 16 cases; T3: 11 cases; T4: 12 cases) performed by the first author (N.K.) at our institution from October 2016 to March 2022. The clinical tumor stage (T1-4) was defined based on the modified Pittsburgh classification, which has been used worldwide for the T stage of external auditory canal squamous cell carcinoma [1].

The clinical NM stage was defined based on the classification adopted for maxillary cancer etc. of the AJCC/UICC staging system (8th edition). We examined the preoperative imaging, surgical approach, margin profile on the pathological specimen, recurrence rate, recurrence site, and prognosis for each case. Our study was conducted according to the guidelines of the Declaration of Helsinki and approved by the ethics committee of Kyushu University Hospital (permit no. 29–43, approval date: 17 April 2017).

The cases we reviewed included two in which the cancer recurred after initial treatment before and comprised 15 males and 25 females (median age: 67.5 years; range: 33–83 years). The median follow-up interval was 25.3 months (range: 6.5–64.3 months). In 11 of the 40 cases (27.5%), cervical lymph node metastasis was diagnosed preoperatively, but none of the patients exhibited distant metastasis. Well differentiated SCC was observed in 26/40 cases (65%), and well to moderately differentiated SCC was observed in 10/40 cases (25%). Lateral TBR (LTBR) was performed in 34 cases and subtotal TBR (STBR) was performed in the remaining six.

**Table 1:** Patient profile.

			n	%	Positive Margin Status (n)
Age	Median(range)		33–83 (67.5)		9
Sex	Man		15	(37.5)	4
	Female		25	(62.5)	5
Side	Right		20	(50.0)	8
	Left		20	(50.0)	1
T	1		1	(2.5)	0
	2		16	(40.0)	1
	3		11	(27.5)	3
	4	Primary	10	(25.0)	3
		Recurrence	2	(5.0)	2
N	−		29	(72.5)	7
	+		11	(27.5)	2
Histology	w		26	(65.0)	8
	w-m		10	(25.0)	0
	other		4	(10.0)	1
Surgery	LTBR		34	(85.0)	5
	STBR		6	(15.0)	4
Preoperative therapy	None		28	(70.0)	7
	IC		8	(20.0)	1
	CRT		1	(2.5)	0
	IC→CRT		3	(7.5)	1

CRT, chemoradiotherapy; IC, induction chemotherapy; LTBR, lateral temporal bone resection; SCC, squamous cell carcinoma; STBR, subtotal temporal bone resection; w, well differentiated; w-m, well to moderately differentiated.

### 2.2. Treatment Strategy

Our treatment policy for EAC-SCC was as follows. The main treatment strategy was to perform en bloc resection for all resectable cases and achieve negative-margin resection. We carefully assessed the extent of tumor extension preoperatively. (Table 2) All surgical procedures were determined based on the direction of tumor extension and included conventional LTBR (cLTBR), extended LTBR (eLTBR) [13], modified STBR (mSTBR) [10], and conventional STBR (cSTBR) [13]. The eLTBR was defined as the cLTBR with superior, inferior, anterior, or posterior extension, as previously reported [13]. The mSTBR was composed of temporal craniotomy rather than temporooccipital craniotomy, and limited posterior mastoidectomy [10]. In STBR, the craniotomy was performed by a neurosurgeon (fourth author, D.K.), and temporal bone cutting was performed by the first author. After resecting the temporal bone, a plastic surgeon performed the reconstruction surgery with a free flap.

Contraindication for surgical intervention was considered when the tumor invasion extended to the internal carotid artery, dura, brain parenchyma, cavernous sinus, nasopharynx, or petrous apex medial to the otic capsule. The inoperable case at initial diagnosis was given curative CRT targeting the primary tumor focus and lymph node. After curative CRT, surgical intervention was considered if the residual lesion was resectable. The RT was administered five days per week (1.6–2.0 Gy/fraction, for a total dose of 60–70 Gy) accompanied by cisplatin (100 mg/m^2^, once every three weeks, 2–3 cycles). Postoperative chemoradiotherapy was administered to patients with positive-margin resections. The TPF regimen (docetaxel, cisplatin, and fluorouracil) was used as induction chemotherapy (5-fluorouracil: 600 mg/m^2^, days 1–5; cisplatin: 60 mg/m^2^/day, day 1; docetaxel: 60 mg/m^2^, day 1) once every three weeks (1–2 cycles). If the tumor was close to the vital structures (internal carotid artery, jugular bulb, sigmoid sinus, posterior and middle fossa dura, and petrous apex) and tumor growth during the waiting period before scheduled surgery could prevent a safe resection margin on preoperative imaging, induction chemotherapy was considered. Induction chemotherapy or preoperative CRT was selected to reduce the tumor size before surgery.

### 2.3. Histopathological Examination

Consensus regarding the definition of a negative or clear surgical margin is lacking [14,15]. Now, to define the surgical margin, so-called “bread loaf” sectioning and specimen-driven sampling are considered to be more accurate to define the surgical margin [15]. Thus, we applied these two methods to define the surgical margin. En bloc resected specimen, which was decalcified, was sectioned into a few mm in width sections. These short-axis sections were embedded in paraffin (formalin-fixed, paraffin-embedded: FFPE). Within each section, a sample of tissue was sliced to examine the tumor margin. At least three pathologists examined whether the tumor cells affected the resection margin, the importance of which was reported by Mazzoni et al. [16]. Perineural (pn), vascular (v), and lymphatic duct (ly) invasion were examined and categorized on a scale of 0 to 3 (0: none, 1: weak, 2: moderate, 3: strong). The results of postoperative pathological examination are listed in Table 2.

### 2.4. Statistical Analysis

Survival rates (disease-specific, overall, and disease-free survival rates) were calculated according to the Kaplan–Meier method. A log-rank test was used to compare the survival distribution between groups. The relationship between disease-free survival and the anatomical factors was examined using a univariate Cox proportional hazards model, JMP 17.0 software (SAS Institute, Cary, NC, USA) was used for statistical analysis, and *p* < 0.05 indicated statistical significance.

## 3. Results

### 3.1. Impact of a Positive-Margin Resection

Nine of the forty cases had positive surgical margins (22.5%; Table 2). Two were recurrent cases and had already received chemoradiotherapy at former hospitals, so they could not receive postoperative chemoradiotherapy despite the identification of positive surgical margins. Two of the cases had no recurrence during the observation period following surgery. Adjuvant postoperative chemoradiotherapy was performed in six cases of the seven cases for which this was the initial treatment. For case 20, instead of cisplatin, the RT (total 60 Gy) was accompanied by oral S-1 (oral fluoropyrimidine) because the patient was aged >80 years. The S-1 was administered orally at 80 mg/m^2^/day for 28 consecutive days, followed by a 14-day rest period while the RT was ongoing. Case 34 received definitive RT without chemotherapy after her surgery because she also had chronic renal disease (stage 3).

We observed postoperative lymph node metastasis and distant metastasis in the cases in which perineural, vascular, or lymphatic duct invasion was found on postoperative pathological examination (Table 2). In addition, three cases in which no vascular, lymphatic duct, or perineural invasion was observed exhibited local recurrence. Only two cases (24 and 26) did not exhibit postoperative recurrence. In case 26, both the anteromedial and posterior surgical margins were affected by the tumor, but no postoperative recurrence was observed following postoperative adjuvant chemoradiotherapy. In case 24, during the mastoidectomy, the tumor was exposed on the posterior aspect, resulting in the positive-margin resection. However, sufficient bony resection was performed. Postoperative pathological examination revealed perineural invasion, thus we administered postoperative chemoradiotherapy.

**Figure 1:**
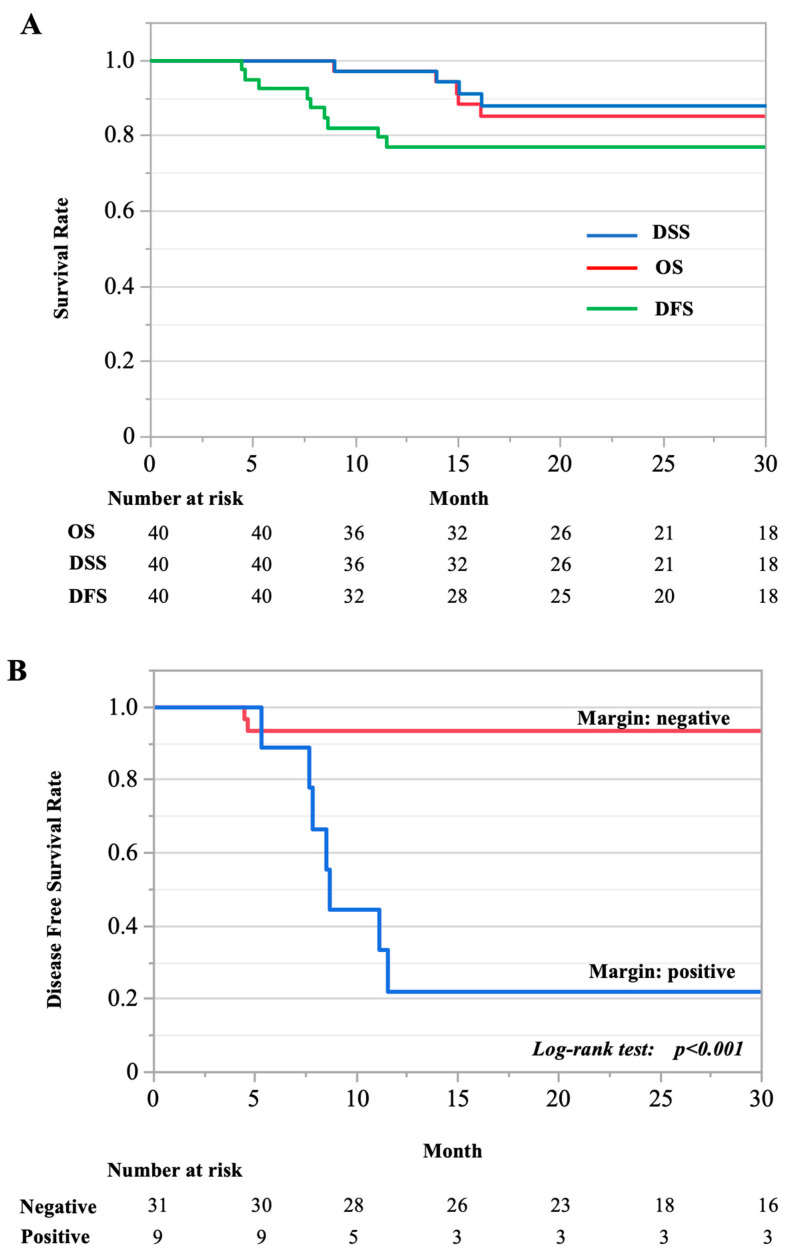
Kaplan–Meier curve. (**A**) Two-year overall, disease-specific, and disease-free survival rates for all cases reviewed. DFS, disease free survival; DSS, disease specific survival; OS, overall survival. (**B**) The survival of two groups compared: cases with negative- and positive-margin resections.

The two-year overall, disease-specific, and disease-free survival rates for all 40 cases with surgical intervention were 85.2%, 88.85%, and 76.96%, respectively (Figure 1A). En bloc resection with a negative margin significantly improved prognosis (*p* < 0.001; Figure 1B).

In the cases with positive-margin resections, it is possible that the preoperative diagnosis underestimated the extent of the tumor [17]. In the present retrospective review, we concluded that the tumor had in fact extended to the parapharyngeal space and the jugular foramen in cases 20 and 40, respectively (Table 2).

Univariate analysis identified T4 status and positive margin status as significant predictors of poor prognosis (Table 3).

### 3.2. Illustrative Cases

#### 3.2.1. Case 20

**Figure 2:**
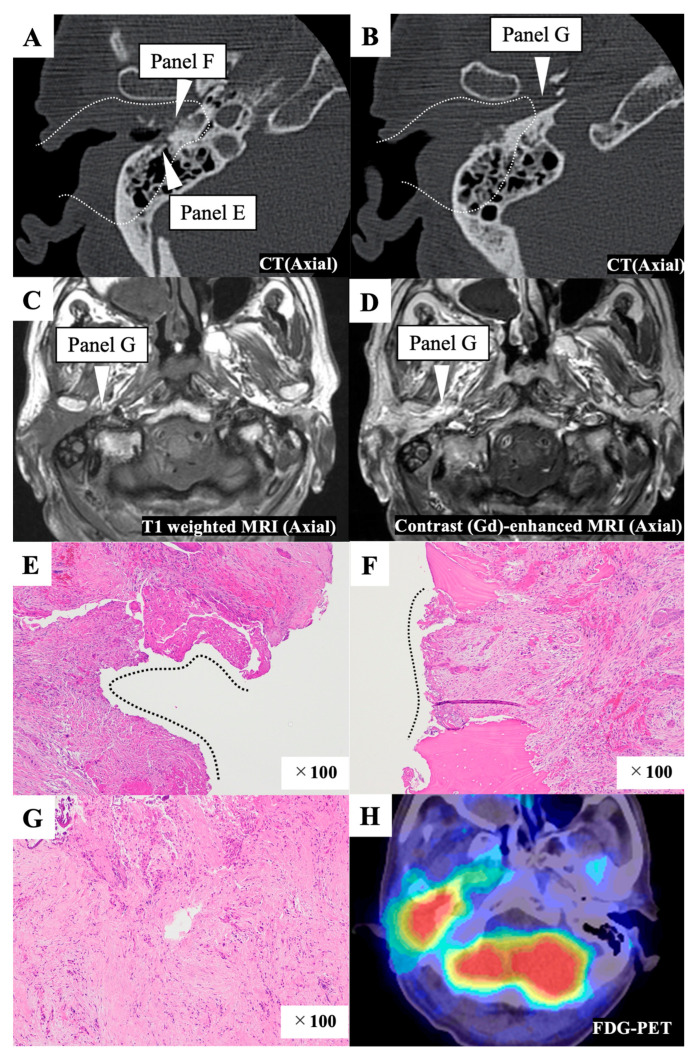
Representative case 20. (**A**,**B**) Preoperative enhanced computed tomography (CT) image. The white dotted line indicates the resection line. The white triangles indicate the areas affected by the tumor, as observed in the postoperative pathological examination. The white dotted line indicates the resection line. (**C**,**D**) Preoperative T1 weighted (**C**) and contrast-enhanced (**D**) magnetic resonance imaging (MRI) images. The white triangle shows that the tumor extends medially toward the parapharyngeal space and indicates the areas affected by the tumor, as observed in the postoperative pathological examination. (**E**) The posterior margin was affected by the tumor, as observed in the postoperative pathological examination. Black dotted line indicates the surface of the specimen affected with the tumor. (**F**) The medial margin was affected by the tumor, as observed in the postoperative pathological examination. Black dotted line indicates the surface of the specimen affected with the tumor. (**G**) Tissue from the parapharyngeal space was sampled for pathological examination after temporal bone resection, and this sample was found to be infiltrated with tumor cells. (**H**) An F-18 fluorodeoxyglucose–positron emission tomography (FDG-PET)/CT scan shows local recurrence six months after the initial treatment.

Preoperatively, the tumor had invaded the anterior and inferior bony wall and extended into the glenoid fossa (Figure 2A,B). It was revealed that tumor had extended medially toward the parapharyngeal space when we examined the preoperative images after surgery (Figure 2C,D). For this patient, we selected anteriorly eLTBR combined with capsulectomy of the temporomandibular joint, superficial parotid gland resection, and neck dissection (IIa,IIb). The tumor was exposed on the specimen’s posterior surface during the mastoidectomy (Figure 2E). The postoperative pathological examination revealed that the posterior and medial margins were affected by tumor cells (Figure 2E,F). After removal of the temporal bone, tissue in the parapharyngeal space was sampled for pathological examination, and this sample exhibited tumor-cell infiltration (Figure 2G). Postoperative chemoradiotherapy was applied, but local recurrence was observed five months after treatment (Figure 2H).

#### 3.2.2. Case 11

**Figure 3:**
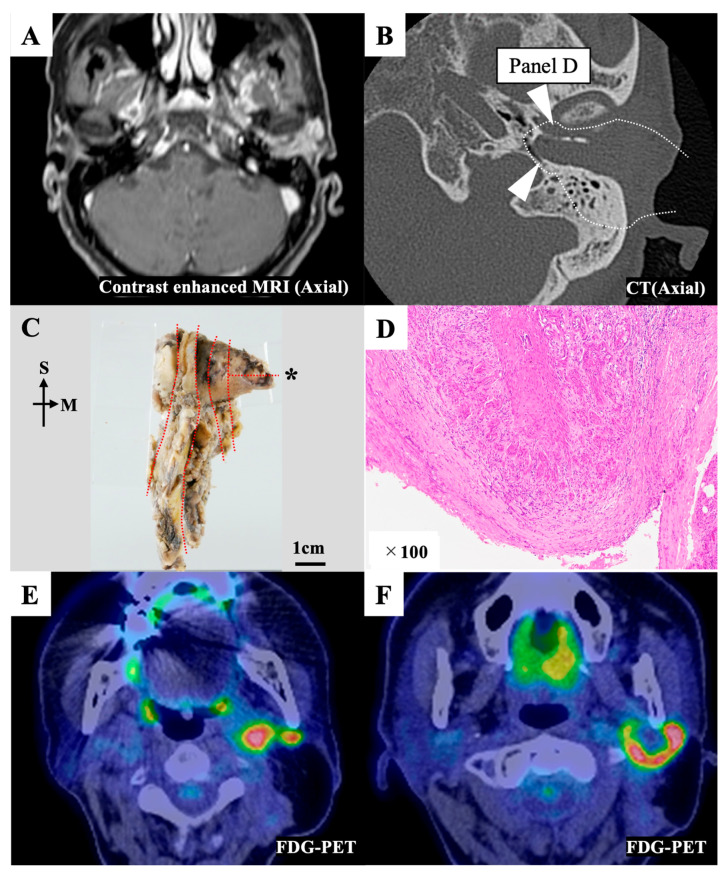
Representative case 11. (**A**) Preoperative contrast-enhanced magnetic resonance imaging (MRI) image. The tumor filled the external auditory canal. (**B**) Preoperative computed tomography (CT) image. The white dotted line indicates the resection line. The white triangles indicate the areas affected by the tumor, as observed in the postoperative pathological examination. (**C**) The en bloc resected specimen was divided into sections (red dotted line) for the pathological examination. The pathological image on the section with an asterisk (*) is shown in Figure 3D. (**D**) The anteromedial margin of section D was affected by the tumor. (**E,F**) An F-18 fluorodeoxyglucose–positron emission tomography (FDG-PET) CT scan showing multiple lymph node recurrence in the infratemporal and retromandibular spaces seven months after the initial treatment. M, medial; S, superior.

On the preoperative image, the bony wall of the EAC had not been destroyed, so this case was diagnosed as T2 (Figure 3A,B). However, we suspected multiple lymph node metastases preoperatively. Thus, for this patient, we selected cLTBR combined with total parotid gland resection and neck dissection (II, III). The postoperative pathological examination (Figure 3C) showed that the surgical margin of the resected specimen’s anteromedial region was almost affected by tumor cells (Figure 3D). After discussion with the pathologist, it was concluded that it would be better to treat it as a positive margin. Tumor cells were exposed on the posteromedial surface of the specimen because the distance between the jugular bulb and the EAC infilled with the tumor was extremely short (Figure 3B). Thus, part of the tumor was exposed during the drilling of the bone between the jugular bulb and the posterior edge of the tympanic membrane. The postoperative pathological examination also revealed that this patient had another nodule in the parotid gland, which was apparently not lymphatic node metastasis. It was diagnosed as a skip lesion of the local tumor (multifocal tumor). This patient exhibited postoperative lymph node recurrence in the infratemporal and retromandibular spaces (Figure 3E,F).

#### 3.2.3. Case 32

**Figure 4:**
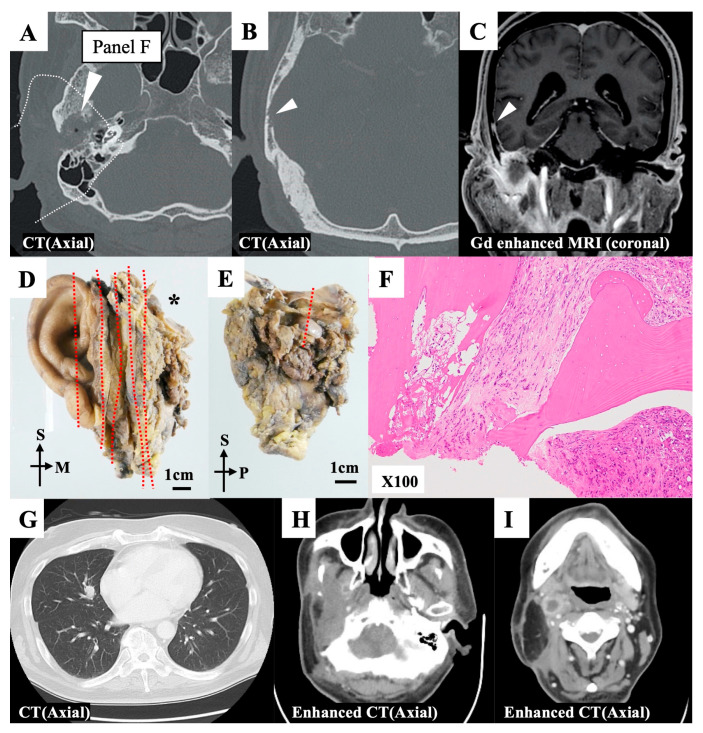
Representative case 32. (**A,B**) Preoperative axial computed tomography (CT) images. The squamous part of the temporal bone exhibited bone absorption (white triangles). (**C**) The nodule was found in the squamous part of the temporal bone, and it continued with dural enhancement of the middle cranial base (white triangle). (**D**) The en bloc resected specimen was divided into sections (red dotted line) for the pathological examination. The divided ection with an asterisk (*) is further examined. (**E**) Section with asterisk in Figure 4D was additionally divided at a red dotted line for the pathological examination. (**F**) The superoanterior margin on the divided surface at a red dotted line in Figure 4E was affected by the tumor. (**G**,**I**) A CT scan revealed a distant metastasis in the right lobe of the lung (**G**) Enhanced CT scans revealed multiple lymph node recurrence in the infratemporal (**H**) and retropharyngeal (**I**) spaces. M, medial; P, posterior; S, superior.

Preoperatively, the tumor had extended into the middle cranial base, accompanied by the destruction of the bony canal of the EAC; however, the jugular fossa and carotid canal were intact (Figure 4A–C). Preoperative contrast-enhanced magnetic resonance imaging (MRI) revealed that the middle cranial base dura was enhanced, reaching into the nodule of the squamous part of the temporal bone (Figure 4C). The tumor was located in the middle cranial base and extended into the glenoid fossa and parotid gland. We performed mSTBR combined with resectioning the squamous part of the temporal bone. The pathological examination (Figure 4D,E) revealed that the anterosuperior margin of the resected specimen, which corresponded to the anteromedial drilling line of the middle cranial base, harbored tumor cells (Figure 4F). However, anteriorly sufficient bony drilling of the middle cranial base had been performed, and a residual tumor was unlikely at the surgical site. Further, we observed no continuity between the nodule in the squamous part of the temporal bone and the middle cranial base. This suggested that the nodule was a distant bone metastasis. The postoperative pathological examination revealed the presence of vascular and lymphatic duct invasions, and we did also observe a distant bone metastasis. These findings provided the rationale for adjuvant chemoradiotherapy. However, this patient exhibited postoperative lymphatic node metastasis and distant metastasis of the lung (Figure 4G–I).

## 4. Discussion

A negative-margin resection of EAC-SCC offers the best prognosis, and this finding is globally accepted. However, even if one carefully assesses the extent of tumor extension preoperatively and selects an appropriate surgical procedure for each case, cases with a positive margin still occur. To date, no literature has focused on cases with positive-margin resection. In this study, we examined the impact of positive surgical margins in resection of EAC-SCC based on pre- and postoperative radiological examination and postoperative pathological examination. In our dataset, the rate of positive-margin resection was 22.5%. The survival rate for cases with positive-margin resections is far worse than that for cases with negative-margin resections. Further, most recurrences following surgical intervention occurred in less than 12 months.

Our results suggest that a positive-margin resection of EAC-SCC worsens the prognosis. The cases with positive-margin resections were more prone to recurrences even with the addition of postoperative adjuvant chemoradiotherapy. In our dataset, only two of the nine cases with positive-margin resections had no recurrences after treatment. In four cases, lymph node metastasis and distant metastasis were observed after treatment. It is difficult to conclude that local recurrence could have been prevented in these cases, because the metastasis may well have occurred before local recurrence became apparent. In our case series, when vascular, lymphatic duct, or perineural invasion was found in the postoperative pathological examination, it had generally occurred via lymphatic or distant metastasis, as has been mentioned previously [18]. Even if these pathological findings were not found, local recurrence was found in three of four cases. Furthermore, in two of the cases with local recurrences, inadequate evaluation of preoperative images led to the positive-margin resection: we confirmed that tumor extension into the parapharyngeal space (case 20) and jugular foramen (case 40) had existed preoperatively.

The efficacy of postoperative radiotherapy for cases with positive margins remains controversial [1,8,9,11,12,13,18]. Shinomiya et al. reported that 4 in 33 patients with early-stage EAC-SCC (T1 or T2) resulted in positive margin resection and 3 of them who underwent postoperative radiotherapy were alive without recurrence during the observation period [19]. Furthermore, they have not recommended postoperative radiotherapy for cases with peri-neural invasion, vascular invasion, or bone invasion but almost all patients have not had local recurrence [19]. Yin et al. reported that six cases with advanced staged EAC-SCC were treated and two cases with positive margin resection died of disease or other cause. They concluded that patients with positive margin, perineural invasion, and T4 stage carry poor prognosis and postoperative radiotherapy is effective in T2-3 disease [20]. In our dataset, cases with positive margin resection were pathologically advanced (T3 or T4), and five in nine cases with positive margin resection had either vascular, lymphatic duct, or perineural invasion. Combinations of these factors may have led to poor control even with postoperative treatment. One of our concerns is the impact of tumor malignancy itself. It is difficult to conclude from this small number of cases whether the high grade of a tumor leads to positive resection margins or whether the positive margin itself has a critical impact on patient prognosis regardless of tumor grade. Generally, the invasive tumor front is the primary site of tumor progression and metastasis. The tumor front encompasses a dynamic process of de-differentiation of the EAC-SCC. Tumor budding or poorly differentiated clusters are found in EAC-SCC specimens and are associated with a poor prognosis [21,22]. Tumor budding is thought to represent the active invasive form of a malignant tumor and occurs as a result of the isolation and mobilization of tumor cells from the main mass during the early stages of tumor invasion [23]. Okado et al. found tumor budding on the invasive front in 71.7% of EAC-SCC cases in the biopsy samples, and the tumor budding cells were positive for laminin-5-γ2 [22]. Further, Miyazaki et al. reported that poorly differentiated clusters on the invasive front were associated with a poor prognosis, and an immunohistochemical study showed that expression of tumoral emmprin was associated with high-grade tumor budding [24]. Emmprin plays a role in the tumor–stroma interaction at the invasive front of a tumor [25,26], and the role of both laminin-5-γ2 and emmprin in the epithelial–mesenchymal transition (EMT) has been proven [27,28]. Tumor cells can develop therapeutic resistance due to EMT-induced antiapoptotic abilities, improved DNA-damage repair, and a modified drug-metabolism pathway [29]. Distinct EMT processes have been observed in chemotherapy- or radiation-resistant tumor cells [30,31,32]. These findings partially explain why a positive-margin resection leads to a poor prognosis and high rates of recurrence and resistance to postoperative adjuvant therapy: the tumor cells at the invasive front, which are of a relatively aggressive nature, tend to be left in the patient.

In treating EAC-SCC, it is one of the recognized pieces of evidence globally that a negative-margin resection can offer the best prognosis. Therefore, maximum efforts should be made to achieve a negative-margin resection. Based on the characteristics of this disease, three points should be considered preoperatively. First, determining whether the tumor extends into the parapharyngeal space and infratemporal fossa preoperatively is crucial. In case 20, the tumor extended into the parapharyngeal space. A CT image revealed the loss of fat density due to tumor extension. We should therefore have performed anteriorly and inferiorly eLTBR, meaning that the bone-cutting line should have been set posterior to the base of the styloid process. Second, accurate assessment of the extent of the tumor based on preoperative image is more difficult in recurrent cases than in the primary tumor. In other words, accurately determining whether areas of reduced bone density are caused by tumor invasion or by inflammation or post-treatment effects is difficult. In such cases, surgical intervention should be carefully considered, especially if such findings are seen around the jugular foramen or the internal carotid artery canal. In case 40, the bone density of the jugular fossa was reduced, and we performed an mSTBR. During the surgery, a tumor extension into the jugular fossa was macrosopicaly found. When surgical intervention is considered for recurrent cancer, the patient should be fully informed before deciding whether to opt for salvage surgery or other therapy, such as immune checkpoint inhibitors. Third, the presence of occult parotid involvement should be assessed. Typically, T1 and T2 tumors are considered to be early stage, and it is assumed that they are still limited to the canal. However, there is a possibility that the tumor, especially if it is located in the cartilaginous portion or anterior wall of the EAC, may extend into the parotid gland despite no finding indicating this [33,34]. Based on the literature, optimal resection of the parotid gland is recommended if an early-stage tumor is located in the cartilaginous portion or anterior wall of the EAC [18,34]. Fortunately, none of the cases in our series had positive-margin resection as a result of occult parotid involvement because we always performed partial or superficial parotidectomy combined with TBR if the tumor was located on the anterior or inferior wall, even for early-stage cases. To avoid unnecessary surgical intervention, which can lead to a positive-margin resection, improvement of the current staging system should be considered. With respect to the treatment of EAC-SCC, the problem with the staging systems must be considered [3,4,35]. In general, T1/T2 and T3/T4 tumors have been considered early and advanced, respectively. The outcomes for T3 tumors have improved with the ongoing development of treatment strategies [5,6,7,36], but the prognosis for T4 cases remains poor [37,38]. The modified Pittsburgh classification system may not accurately predict the prognosis of advanced cases because it assigns resectable and unresectable tumors to the same category (T4). Shinomiya et al. [4] focused on this classification issue. They proposed a staging scheme that divides T4 cases into two categories according to the prognostic factors of brain invasion, internal carotid artery invasion, and internal jugular vein invasion. Invasion of the internal carotid artery or brain means that resection without disastrous postsurgical complications is impossible. In our previous research, the prognosis of advanced resectable cases has been significantly better than that of advanced unresectable cases [13,37]. In addition, among cases in which surgical intervention is performed, those with positive margins have a poorer prognosis [13,37]. We therefore propose that T4 cases are divided into two subcategories: unresectable and resectable cases.

For inoperable disease, concurrent chemoradiotherapy with platinum agent represented by cisplatin was the standard treatment. Induction chemotherapy theoretically could reduce the tumor volume and raise the possibility of making the tumor respectable, and hopefully leads to eradicating micrometastases and reducing locoregional failure [39]. However, the role of the preoperative treatment represented by induction chemotherapy on the surgical margin or respectability remains unclear. The main goal of the surgical intervention for external auditory canal carcinoma is to achieve a margin-negative resection. Several reports from India on unresectable oral cavity cancer, not external auditory canal carcinoma, showed that induction chemoradiotherapy was effective in reducing the tumor and increasing the rate of resectable cases [39,40,41]. The resectability after induction chemoradiotherapy was reported to be about 20–40% [39,40,41]. However, these were retrospective studies and did not harbor a high level of evidence [39,40,41]. Patil et al. reported that induction chemotherapy for unresectable oral cavity cancer effectively reduced the tumor volume, and 44.7% of patients could undergo surgery resulting in R0 resection [39]. For external auditory canal carcinoma, there is one paper from India reporting that one in four cases of unresectable rumor could undertake surgical resection after the tumor reduction by induction chemotherapy [42]. However, at present, there is no clear evidence of the effects of induction chemoradiotherapy for EAC-SCC on resectability and resection margins. Among 40 cases in our study, 28 cases did not undertake preoperative treatment, and 11 cases undertook preoperative treatment, including induction chemotherapy. However, there was no significant difference in the rate of the positive margin status between the two groups. We believe that it is worth further investigation.

Our study had three limitations. First, it was a single-center retrospective study. Second, the number of cases examined was small because of the extreme rarity of EAC-SCC. Third, the T stage of our cases was diverse, and preoperative treatment and the surgical difficulty was different among cases. Hence, drawing comprehensive conclusions regarding the impact of a positive-margin resection based only on the present study is difficult. Further research is thus required in studies involving larger cohorts and multiple centers.

It is clear that negative-margin resection is the most reliable treatment strategy for improving the prognosis for EAC-SCC. However, based on our data, if postoperative pathological examination confirms a positive-margin resection, those cases may have recurrence even with postoperative adjuvant therapy. In such cases the risk of recurrence is higher, and we would recommend closer imaging follow-up than usual, and efforts should be made to detect recurrence as early as possible, to facilitate the introduction of immune checkpoint inhibitors or other chemotherapies as soon as possible.

## 5. Conclusions

Negative-margin resection of EAC-SCC provides the best prognosis. However, once positive-margin resection is confirmed, we need to keep in mind the possibility of recurrence even with the addition of postoperative adjuvant chemoradiotherapy. In particular, when vascular, lymphatic duct, or perineural invasion is found in the postoperative pathological examination, lymphatic or distant metastasis tends to occur before local recurrence in cases with positive-margin resections.

## Figures and Tables

**Table 2:** Cases with positive surgical margins.

#	Treatment	Surgical Type	TNM Stage				Pathological Evaluation (v, ly, pn)	Preoperative assessment of tumor extension																		Margin	Rec.
			Clinical		Pathologic			External Auditory Canal	Full Thickness Invasion	Middle Ear	Mastoid Fluid	FN Paralysis	Fallopian Canal	Glenoid Fossa	Mandibular Condyle	Stylomastoid Foramen	Parotid Gland	Pterygoid Muscle	Middle Cranial Fossa	Jugular Process	Jugular Foramen	Eustachian Tube	Ossicles	Skin	Parapharyngeal Space		
			T	N	T	N																					
11	Surgery *	cLTBR	2	2 b	m3	0	1, 0, 1	+																		+	N
20	Surgery *	a–eLTBR	3	0	3	0	0, 0, 0	+	+		+		+	+											★	+	T
24	Surgery *	a–eLTBR	3	0	3	1	0, 0, 1	+	+	+	+			+		+										+	
26	Surgery *	cLTBR	3	1	3	0	0, 0, 0	+	+																	+	
32	IC + CRT→Surgery	mSTBR	4	0	y4	0	2, 0, 2	+	+					+			+		+							+	N,M
33	IC→Surgery *	a/i-eLTBR	4	0	y4	3 b	1, 1, 0	+	+					+											+	+	N,M
34	Surgery *	cSTBR	4	0	4	3 b	0, 0, 0	+	+	+	+		+	+	+	+	+	+	+	+	+	+	+	+	+	+	T
39	Surgery	mSTBR	r4	0	r3	0	0, 0, 2	P.O.	P.O.	P.O.	+	+	+				+						P.O.	+		+	N
40	Surgery	mSTBR	r4	0	r4	0	0, 0, 0	+	+	+	+										★	+	+			+	T

a, anteriorly; c, conventional; CRT, chemoradiotherapy; e, extended; i, inferiorly; FN, facial nerve; IC, induction chemotherapy; N, lymph-node metastasis; ly, lymphatic invasion; M, distant metastasis; m, modified; pn, perineural invasion; P.O., postoperative state; Rec., recurrence, v, vascular invasion; T, local recurrence. The black star indicates the site where the preoperative diagnosis underestimated the extent of the tumor. Prefix “m”, “y”, and “r” on TNM classification indicates multifocal tumor, the state after chemoradiotherapy, or recurrent tumor, respectively. Asterisk shows the addition of the postoperative (chemo-)radiotherapy.

**Table 3:** Patient factors predictive of disease-free survival (univariate analysis).

		HR	95% CI	*p* Value
Age	(65≤/65> years)	0.37	0.10–1.34	0.118
Sex	(Male/Female)	0.68	0.17–2.63	0.575
Side	(Rgt/Left)	4.61	0.98–21.75	0.054
Nodal involvement		0.60	0.13-2.83	0.52
Margin status	(Positive/Negative)	16.45	3.90–111.69	0.0006
T stage	(T4/T1-3)	7.38	1.89–28.77	0.004

CI = confidence interval; HR = hazard ratio.

## Data Availability

The authors confirm that the data supporting the findings of this study are available within the article.
